# Hospital Finance

**Published:** 1922-09

**Authors:** 


### HOSPITAL FINANCE.

### HOSPITALS OF LONDON COMBINED APPEAL: FIRST DISTRIBUTION.

A SPECIAL MEETING of the President and General Council of King Edward's Hospital Fund for London was held at the House of Lords on August 1, 1922, to authorise an interim distribution on account of the proceeds of the Hospitals of London Combined Appeal. Mr. M. C. Norman, Governor of the Bank of England, presided. The appointment of Lord Bearsted as a member of the Council was reported.

Sir Alan Anderson, Chairman of the Organising Committee of the Appeal, thought the Council would be interested to hear, at the end of the first stage of the appeal, how closely the amount in hand approached what was expected. Before they started in April, Mr. Shrapnell-Smith hoped to get in £300,000 by July 31. Mr. Shrapnell-Smith now reported that, including the money advised and intimated, but not yet received at headquarters, he hoped slightly to exceed the £300,000. Of this, £271,000 was actually in hand, and large sums were coming in every day. When allowance was made for expenses incurred in advance of the next stage of the appeal, it appeared that the expense to date was about £30,000, a much lower figure than the Appeal Committee had been warned to expect. The free grant of space on hoardings, by the Office of Works, the London County Council, and various boroughs, had made it possible to reach the public by poster at about one-fifth the ordinary cost of such advertisement.

The work entailed could not have been done without the special advantages which the King's Fund had, and which no other appeal could have had. The support given by the Royal Family was of immense value. The Prince of Wales had consented to sign souvenir receipts for all who gave £1,000 or over; about 65 such receipts had now been earned. To the funds opened by the Lord Mayor, and the Mayors and Chairmen of Local Councils, they were indebted for about £115,000. The students had been of enormous use, and had put life into everything they touched. At the centre, Sir George Lawson Johnston and Major Harold Wernher were responsible, with Mr. Shrapnell-Smith (Director General), Mr. Macrae (Finance Director), and Mr. Elliot (Secretary), for the successful co-operation of so many hospitals and organisations scattered over so wide an area; while the Council would also wish to thank the Trustees of the Appeal, the Lord Mayor, Lord Hambleden, Lord Revelstoke, and the Governor of the Bank of England, for their advice and help.

In the autumn, the chief elements of the appeal, apart from care of money boxes, badges and stamps, would be the educational campaign which Lord Burnham and Professor Winifred Cullis were organising, the theatrical campaign under Sir Gerald du Maurier and Mr. George Grossmith, and the appeal to domestic servants. Many local efforts remained to be pressed home, and so far as possible these would be undertaken by the appropriate key hospital and mayoral committee. Most important of all was the Hospital Savings Association, of which Lord Hambleden was Chairman, which was intended to bring regular weekly contributions from wage-earners to the hospitals. It was really to give the hospitals time to get those regular weekly contributions organised that the Combined Appeal was started. The Organising Committee had every wish to retire from business as soon as possible, but the Council would appreciate, from this sketch of their existing activities, that it was necessary to carry on to a certain extent during the autumn. The temporary Government grant to hospitals had been subject to a fundamental condition that an equal sum should be raised from the public. The appeal had already succeeded in raising as much "new money" as the maximum amount of Government money at present available for London hospitals, and the newspapers and mayoral and other committees had been informed that the Government could no longer give "pound for pound" to balance new money.

The Chairman of the Management Committee, Lord Stuart of Wortley, presented a report which stated that, under the terms of the circular of March 20, 1922, inviting hospitals to co-operate, each hospital co-operating in the appeal, on the conditions set out in the scheme, would receive a share of the proceeds based, *inter alia,* on the number of occupied beds and out-patient attendances, and on the evidence as to general efficiency, financial needs, and efforts made to assist the appeal. A full examination of the circumstances of the co-operating hospitals would necessarily take a good deal of time. The final allocation could not be settled until this examination was completed, and until more was known as to the ultimate proceeds of the appeal and as to the basis on which the Voluntary Hospitals Commission would distribute the balance of the Government grant available for London hospitals. It appeared to the Management Committee that the largest sum that should be paid out on the information at present available was £150,000. In these circumstances, the Management Committee recommended:—

> That the King's Fund should pay to the co-operating hospitals cheques on account, on some date between August 1 and August 15, amounting in all to £150,000; and that a further payment on account should be made to each hospital after the holidays as soon as sufficient further information became available.

### DISTRIBUTION OF PROCEEDS.

The following sums have since been paid on account:—

Acton Hospital, £235; All Saints' Hospital, £405; Anti-vivisection Hospital, £330; Baby Clinic and Hospital (Kensington), £150; Beckenham Cottage Hospital, £160; Belgrave Hospital for Children, £525; Blackheath and Charlton Hospital, £160; Bolingbroke Hospital, £415; Brentford Cottage Hospital, £30; British Hospital for Mothers and Babies, Woolwich, £285; Cancer Hospital, £765; Canning Town Women's Settlement Hospital, £255; Central London Ophthalmic Hospital, £400; Central London Throat and Ear Hospital, £400; Chelsea Hospital for Women, £500; Cheyne Hospital for Children, £400; City of London Hospital for Diseases of the Chest (Victoria Park), £1,745;

City of London Maternity Hospital, £590 ; Clapham Maternity Hospital, £300 ; Dreadnought Hospital (Seamen's), £3,310 ; East End Mothers' Lying-in Home, £370 ; East Ham Hospital, £110 ; East London Hospital for Children, £2,200 ; Elizabeth Garrett Anderson Hospital, £1,150 ; Eltham and Mottingham Cottage Hospital, £95 ; Evelina Hospital, £680 ; Finchley Cottage Hospital, £190 ; Florence Nightingale Hospital for Gentlewomen, £350 ; General Lying-in Hospital, £350 ; German Hospital, £1,040 ; Grosvenor Hospital for Women, £285 ; Guy's Hospital, £8,950 ; Hampstead General and North-West London Hospital, £1,800 ; Hendon Cottage Hospital, £265 ; Hornsey Cottage Hospital, £235 ; Hospital for Consumption, Brompton (including Sanatorium at Frimley), £3,460 ; Hospital for Epilepsy and Paralysis, £690 ; Hospital for Sick Children, £3,600 ; Hospital for Women (Soho Square), £540 ; Hospital of St. John and St. Elizabeth, £890 ; Hostel of God, £145 ; Hostel of St. Luke, £80 ; Infants' Hospital, £350 ; Italian Hospital, £220 ; Jewish Maternity Home, £85 ; Kensington, Fulham and Chelsea General Hospital, £255 ; Kensington Dispensary and Children's Hospital, £80 ; King Edward Memorial Hospital (Ealing), £365 ; King's College Hospital, £5,500 ; London Fever Hospital, £955 ; London Homœopathic Hospital, £1,355 ; London Hospital, £10,400 ; London Jewish Hospital, £315 ; London Lock Hospital, £1,380 ; London Temperance Hospital, £1,500 ; Maternity Charity and District Nurses' Home, Plaistow, £1,565 ; Metropolitan Ear, Nose and Throat Hospital, £145 ; Metropolitan Hospital, £2,250 ; Middlesex Hospital, £5,615 ; Middlesex Hospital Cancer Charity, £855 ; Mildmay Memorial Hospital, £185 ; Mildmay Mission Hospital, £640 ; Miller General Hospital for South-East London, £2,200 ; Mothers' Hospital of the Salvation Army, £540 ; National Hospital for Diseases of the Heart, £460 ; National Hospital for the Paralysed and Epileptic, £2,500 ; Nelson Hospital (South Wimbledon), £235 ; Northcourt Hospital and Home for Sick Children (Hampstead), £380 ; North Islington Infant Welfare Wards, £75 ; Paddington Green Children's Hospital, £445 ; Passmore Edwards Hospital for Wood Green, &c., £170 ; Poplar Hospital for Accidents, £1,055 ; Prince of Wales's General Hospital, £2,300 ; Queen Charlotte's Lying-in Hospital, £885 ; Queen Mary's Hospital for the East End, £1,860 ; Queen's Hospital for Children, £2,700 ; Royal Dental Hospital of London, £345 ; Royal Eye Hospital, £560 ; Royal Free Hospital, £5,000 ; Royal Hospital, Richmond, £395 ; Royal London Ophthalmic Hospital, £1,740 ; Royal National Orthopædic Hospital, £2,445 ; Royal Northern Hospital (with which is amalgamated the Royal Chest Hospital, City Road), £5,500 ; Royal Waterloo Hospital for Children and Women, £1,050 ; Royal Westminster Ophthalmic Hospital, £410 ; St. Andrew's Hospital (Dollis Hill), £210 ; St. Bartholomew's Hospital, £7,700 ; St. Columba's Hospital, £300 ; St. George's Hospital, £3,500 ; St. John's Hospital, Lewisham, £1,165 ; St. John's Hospital for Diseases of the Skin, £365 ; St. Luke's Hospital for Advanced Cases, £225 ; St. Mark's Hospital, £325 ; St. Mary's Hospital, £3,350 ; St. Mary's Hospital for Women and Children, Plaistow, £720 ; St. Monica's Home Hospital, £200 ; St. Peter's Hospital for Stone, £385 ; St. Saviour's Hospital for Ladies of Limited Means (Osnaburgh Street), £125 ; St. Thomas's Hospital, £7,700 ; Samaritan Free Hospital for Women, £650 ; Santa Claus Home, £145 ; South-Eastern Hospital for Children (Sydenham), £585 ; South London Hospital for Women, £1,005 ; Stoke Newington Home Hospital for Women, £120 ; Streatham Babies' Hospital, £105 ; University College Hospital, £4,000 ; Victoria Hospital for Children, £1,285 ; Walthamstow, Wanstead and Leyton Children's and General Hospital, £355 ; Weir Hospital (Balham), £195 ; West End Hospital for Nervous Diseases, £670 ; Western Ophthalmic Hospital, £300 ; West London Hospital, £3,150 ; Westminster Hospital, £3,000 ; Willesden Hospital, £630 ; Wimbledon Hospital, £255 ; Winifred House Invalid Children's Convalescent Hospital Home, £120 ; Woolwich and Plumstead Cottage Hospital, £60 ; Woolwich and District War Memorial Hospital, £1,000.

---

Mr. A. H. Leaney, House Governor of the General Hospital, Birmingham, has announced that it is hoped to open all the closed beds at the General and Jaffray Hospitals on October 1. Not only money but nurses are needed, and young men also to help in hospital management.

### UNDERMINING A SATURDAY FUND.

An unfortunate situation has arisen in regard to one of the Hospital Saturday funds in the provinces. It appears that the fund is at a low ebb for two reasons. The different works in the town have started welfare schemes of their own, and apparently all the money collected in these works goes to the support of these schemes, and no " recommends " are available for anyone but their employees. The consequence is that the general public when needing a " recommend " has great difficulty in finding one. The difficulty, to some extent, affects the employees also, because, while the welfare schemes provide for certain needs, none of the schemes covers all the hospitals and institutions. For those institutions outside the scope of the welfare schemes " recommends " have to be sought at the Town Hall ; the general Saturday Fund provides them, but is ill supported because, no doubt, people are unwilling to subscribe to the general fund in addition to the welfare fund centred in their particular workshop. Indeed, according to the secretary of this local Saturday Fund, when the Fund's committee started a penny a week collection at one works, the firm kept the money and applied it to its own welfare fund. There is, then, a clear case of overlapping. The welfare schemes are encroaching on the work of the Saturday Fund, which is the elder organisation, and at least in one case have gone so far as to appropriate subscriptions intended for the Saturday Fund. The remedy lies in the hands of the workers themselves, who should decide whether they prefer a local welfare scheme that only provides " recommends " for some of their needs in sickness, or whether they would rather support the central Saturday Fund whose " recommends " are available for all institutions. There is evidently no room for welfare schemes of this pretension and a central Saturday Fund on traditional lines. We have heard of similar overlapping in recent years since welfare schemes became popular, but this is the most acute example that has come to our notice.

---

### THE FRYATT MEMORIAL HOSPITAL.

The recent opening by Lord Claud Hamilton of the Fryatt Memorial Hospital gave to Harwich and the district the cottage hospital so long desired. The late Dr. Richmond and the trustees of Mr. Whitaker had provided the initial fund, to which was added the sum raised to perpetuate the memory of Capt. Fryatt, who, as we all remember, was officially murdered by the Germans. The proceeds of the sale of Capt. Fryatt's vessel, s.s. "Brussels" (£2,100) was also handed over by the Government to the hospital trustees, and with this a house was bought and converted. Mr. Vincent Brown is the architect. It possesses a men's ward, a women's ward, a children's ward, and two private wards, a theatre and an X-ray room. The alterations and equipment have cost some £5,000, or less than half the total fund raised. The new hospital should relieve the strain upon the Ipswich and Colchester hospitals, and become self-supporting without competing with them. The insufficiency of hospital beds in this country is so serious that the opening of a new hospital, partly endowed, is generally welcome; and from the name which it commemorates—Capt. Fryatt is buried close by—the institution is one of the most interesting additions of the year.

### THE HOSPITAL SATURDAY FUND.

At the annual meeting of the Hospital Sunday Fund, the Distribution Committee reported that the total of the fund amounted to £95,000 (only £5,000 less than last year), which would be distributed among 220 institutions, as compared with 226 in 1921. The principal awards were as follows:—London Hospital, £10,000; Middlesex, £4,250; St. George's, £3,750; King's College, £3,570; Charing Cross, £2,370; Gt. Northern Central, £2,700; Guy's, £4,740; Hampstead and N.-W. London, £1,115; Metropolitan, £1,300; Miller, Greenwich, £1,040; Prince of Wales, Tottenham, £1,230; Royal Free, £2,770; St. Mary's, £2,685; St. Thomas's, £2,860; Seamen's, £1,720; University College, £2,650; Queen Mary's, £1,410; West London, £2,060; Westminster, £2,240; Victoria Park Chest Hospital, £1,000; Brompton, £1,200; Hospital for Sick Children, £1,990; Queen's Hospital for Children, £1,970; Victoria Hospital, £1,125; East London Hospital for Children, £1,150; National Hospital for Paralysis, £1,300; Royal London Ophthalmic, £1,250; Metropolitan Convalescent Institutions, £1,290. Seven and a half per cent. of the total sum available was retained for the purchase of surgical appliances during the year, and 2½ per cent. for district-nursing associations.

A sum of nearly £50,000 is to be spent upon new anatomical and biological departments at Guy's Hospital.

Mr. G. H. Gunning has given a site for a new hospital at Erith, to cost £12,000, of which a third has been conditionally subscribed.

The Islington Borough War Memorial will be an extension of the Royal Northern Hospital by a complete casualty department. The scheme is expected to cost £11,000.

The colliery proprietors in the Wigan Mining district are supplying coal free of charge to the Wigan Infirmary for twelve months. In four months the gift has saved the Infirmary £500.

The Wolverhampton Guardians have either doubled or largely increased their subscriptions to the local hospitals and nursing associations. Their subscription to the General Hospital is now 50 guineas instead of 20 guineas.

A new means of raising money was successfully tried at Oldham Royal Infirmary, which one afternoon was thrown open for public inspection, when the special departments were explained to the visitors. A silver collection was taken at the door; 3,500 people attended, and contributed £150.

The Isle of Arran War Memorial Hospital at Lamlash has been opened by the Duchess of Hamilton. Designed by Mr. Archibald Cook, the hospital, which will be open for all non-infectious patients, including visitors, possesses two wards with four beds apiece, two single wards, a theatre, ten bedrooms, offices, garage, and engine-house. The sum collected is £14,135.

Paying patients are being admitted to the Leeds Union Infirmary in Beckett Street. The condition is that of necessity, which may arise from want of a hospital bed elsewhere or from want of facilities in the patient's own home. The charge varies from £1 per week, according to the patient's ability to pay. Those who can afford a nursing home are not admitted. Each case is considered separately.

In one of the London schools the children, according to an L.C.C. teacher in "The Star," were stimulated to collect money for the hospitals by watching a figure climb a steep track up a high mountain. The track was divided into ten stages, each representing £1. That the top might be reached, a jumble sale was arranged by four classes, and further efforts are planned after the holidays.

